# Rare but Real: Severe Unilateral Macroglossia and Submandibular Sialoadenitis After Skull Base Surgery

**DOI:** 10.7759/cureus.55075

**Published:** 2024-02-27

**Authors:** Frederic Van Havenbergh, Jarne Schepens, Michel Torfs, Tony Van Havenbergh

**Affiliations:** 1 Neurosurgery, Ziekenhuis aan de Stroom (ZAS) Hospitals, Antwerp, BEL; 2 Anesthesiology, Gasthuis Zusters Antwerpen (GZA) Hospitals, Antwerp, BEL

**Keywords:** patient positioning, skull base surgery, sialoadenitis, macroglossia, complications

## Abstract

We present a 43-year-old patient with a left-sided cerebellopontine angle meningioma with extension to the internal acoustic meatus and jugular foramen. The patient underwent a resection using a retrosigmoid approach, which resulted in near-complete tumor removal. Postoperatively, the patient experienced tongue swelling, swallowing difficulties and right-sided subcutaneous swelling, caused by patient positioning and endotracheal tube placement. Imaging showed phlegmonous infiltration of subcutaneous fat tissue with submandibular gland enlargement. The patient's condition gradually improved with conservative management. This case highlights the rare occurrence of combined macroglossia and sialoadenitis after posterior fossa surgery, emphasizing the importance of patient positioning and tube placement.

## Introduction

Cerebellopontine angle tumors often present with a variety of symptoms caused by cranial nerve compression. Surgical treatment in growing lesions is considered best medical treatment, although serious complications occur [1].

Our case report presents an unexpected postoperative complication, starting with tongue swelling and progressing to an impressive enlargement of the submandibular gland with profound swallowing disturbances, leading to prolonged need for intensive care and hospital admittance.

This report aims to underscore the imperative need for heightened vigilance during patient positioning, tube placement, and postoperative management to avert unusual but potentially critical complications in skull base surgery. This case emphasizes the necessity for further investigation and awareness among neurosurgical teams to ensure optimal patient outcomes.

## Case presentation

**Figure 1:**
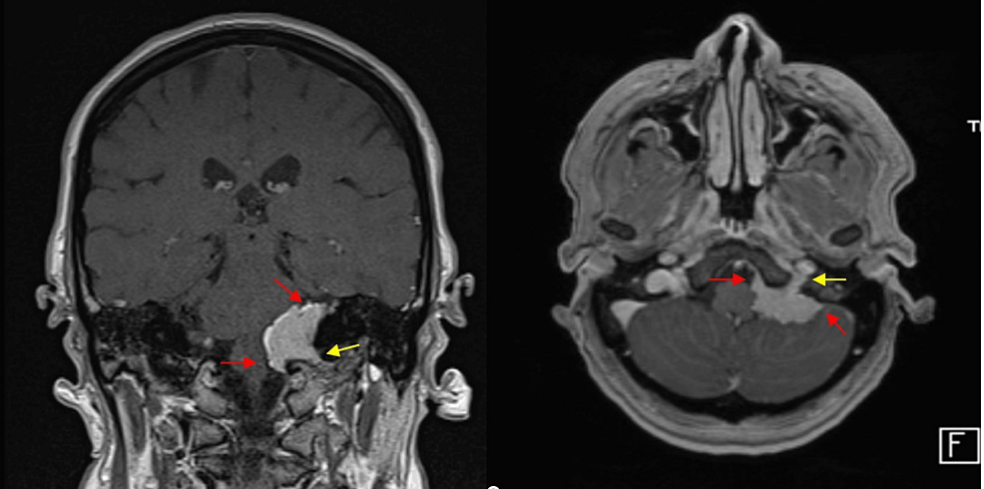
Pre-operative MRI shows a contrast-enhancing lesion in the cerebellopontine angle with a dural tail with brain stem compression (red arrows). An extension to the meatus acusticus internus can be seen (yellow arrows).

We present a case of a 43-year-old patient who presented with tinnitus, hearing loss, disequilibrium, and vague swallowing disturbances. MRI revealed a left-sided cerebellopontine angle tumor with a dural tail and extension to the internal acoustic meatus and jugular foramen with brainstem compression, most likely a meningioma (Figure 1).

**Figure 2:**
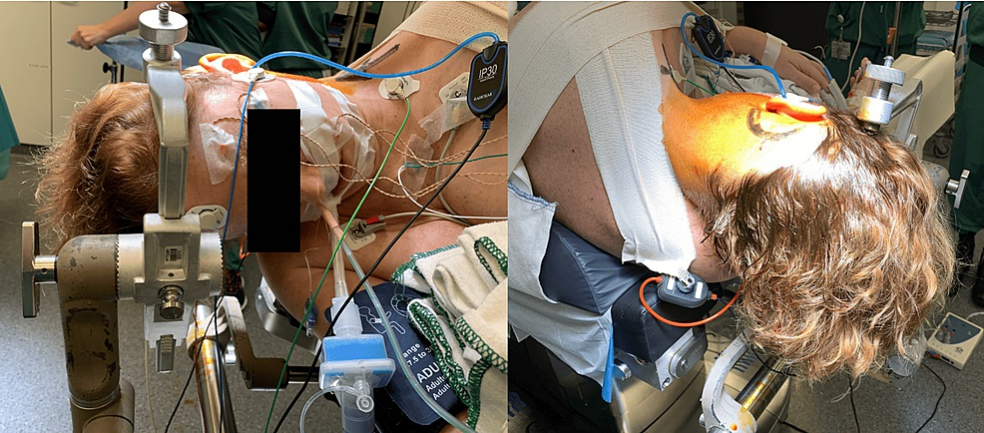
Patient positioning The patient was positioned on the right side. The head is tilted downwards (neck lateroflexion) with minimal downward rotation ("the patient is looking at the floor"). Maximal neck flexion ("chin on chest") creates more working space to approach the cerebellopontine angle. The tube was fixated contralateral to the operative field. The upper shoulder was retracted away from the head.

A resection was performed using a C-shaped retrosigmoid approach in the right lateral decubitus position with the head fixated in the Mayfield clamp. The head is positioned as shown in Figure 2. Mayfield clamp was positioned vertically. An armored endotracheal tube size 7 (with neuromonitoring) was placed before patient positioning and was minimally manipulated after positioning to insert an intraoral bite block. A nasogastric tube was placed. The tube was fixated in the corner of the mouth on the right side. Neuromonitoring of the facial nerve (n. VII), cochlear (n. VIII) and lower cranial nerves (n. IX, X, and XI) was utilized. A near-complete removal of the tumor was achieved, leaving a small part of tumor within the jugular foramen to maintain the function of the lower cranial nerves. The total duration of the procedure, including general anesthesia, was approximately 10 hours.

**Figure 3:**
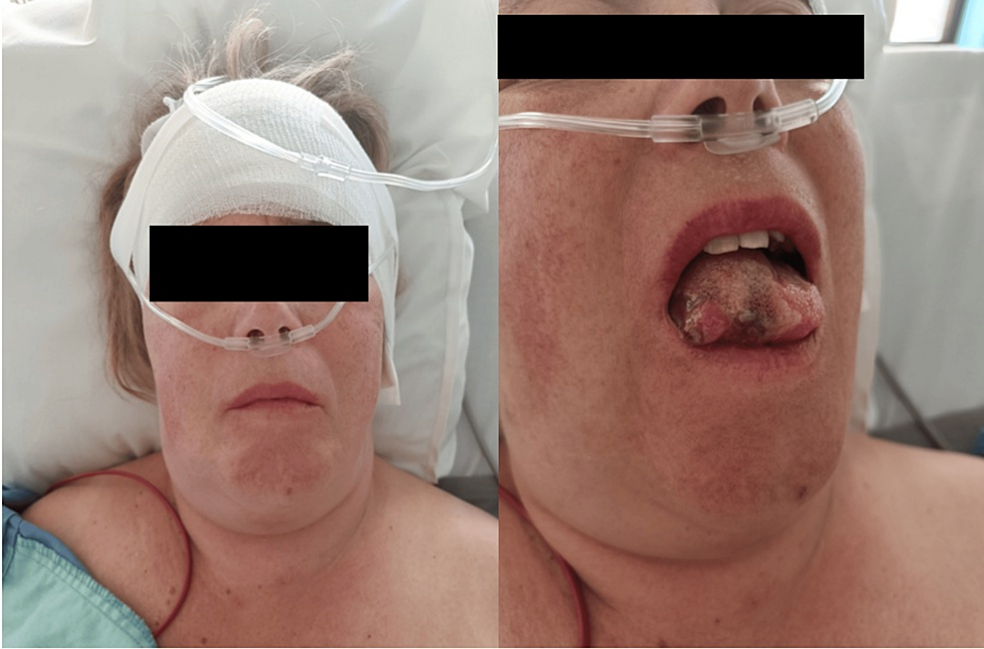
A severe right-sided swelling of the submandibular region can be seen. A profound macroglossia with local necrosis was noted intraorally.

**Figure 4:**
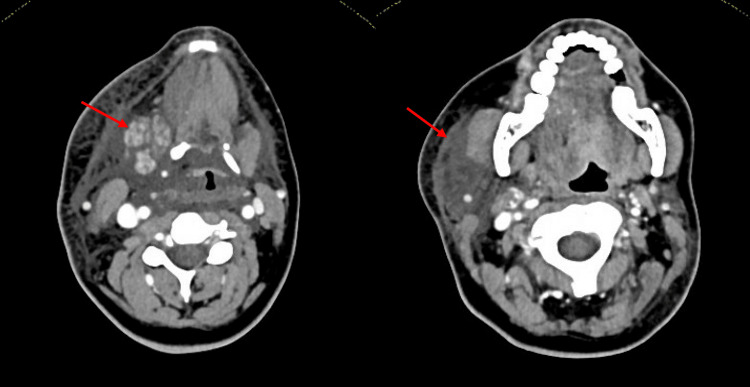
Post-operative CT of the neck region shows a remarkable swelling of the submandibular gland with patchy contrast-enhancing and infiltrations of the subcutaneous tissues.

Postoperatively, the patient experienced a numb and swollen sensation in the tongue, along with swallowing difficulties. No clear mucosal damage was seen in the acute postoperative phase. The swelling gradually increased, and a marked enlargement of the right submandibular gland was observed, at the opposite side of the surgery, as shown in Figure 3. Urgent neck CT with contrast revealed phlegmonous infiltration of the subcutaneous fat tissue and enlargement of the right-sided submandibular gland with limited contrast enhancement (Figure 4). Laryngoscopy confirmed right-sided edematous swelling of the larynx without airway compromise.

On the fifth postoperative day, necrosis of the right side of the tongue was noted, accompanied by rapid shrinking of the laryngeal edema. A nasogastric tube was replaced due to persistent swallowing difficulties without a clear anatomical explanation. After one week, the submandibular gland enlargement gradually diminished, with persisting swallowing difficulties. At three months postoperatively, a remarkable functional improvement was seen, although a discomforting tongue ulcer persisted. Histopathological examination of the resected tumor confirmed the diagnosis of a WHO grade 1 meningioma.

## Discussion

This case report describes the unique presentation of combined macroglossia and sialoadenitis following posterior fossa surgery. Macroglossia, characterized by tongue swelling, has been reported after various types of surgeries, particularly those involving the posterior fossa. Although the exact etiology of postoperative macroglossia remains unclear, several contributing factors have been proposed.

One hypothesis suggests that venous and lymphatic congestion resulting from extreme neck flexion or local mechanical compression could lead to macroglossia [2-8]. Vermeersch et al. [7] described a combination of neck flexion with venous and lymphatic congestion, compression of the tube with oral packing (bite block) at the base of the tongue, and compression of the lingual artery and deep lingual veins, which could provoke macroglossia. The rapid postoperative swelling may be attributed to reperfusion with secondary hyperaemia and capillary leakage [9].

Another potential explanation involves neurogenic mechanisms triggered by manipulation of the brainstem, leading to discharges of sympathetic activity. Moore et al. have described macroglossia resulting from neurogenic factors [10].

In addition to macroglossia, a remarkable enlargement of the submandibular gland was observed on the right side as well. This enlargement developed gradually over the first few hours and is difficult to explain by macroglossia alone. A recent retrospective review by Yagnik et al. [11] reported sialoadenitis as a rare complication after neurosurgical procedures. In this limited series, sialoadenitis typically manifested as gradual enlargement of the parotid or mandibular gland after three to four hours. While 83% of the reported cases were associated with skull-base surgery, no specific positioning (sitting, park-bench, or lateral decubitus) was identified as a consistent factor. It is presumed that prolonged rotation of the neck during surgery and mechanical compression of the gland may cause occlusion of Wharton's duct, leading to the development of sialoadenitis.

Other hypotheses propose local compression of the lingual or facial artery, resulting in ischemia and subsequent reperfusion edema [4,11-14]. Interestingly, a predilection for younger women (<50 years) has been observed, possibly due to larger gland size and lower salivary flow rate [11].

In the present case, a right-sided pressure ulcer was observed after a few days, suggesting a local compression, likely caused by a combination of the armored tube, bite block, tube positioning contralateral to the craniotomy and manipulation of the tube after positioning in the lateral decubitus position. In cerebellopontine angle surgery, the patient is usually positioned in laterale decubitus or park bench positioning, with neck (latero) flexion and rotation to improve visualisation of the cerebellopontine angle (Figure 2). This positioning can lead to a venous congestion at the opposite side. The combination of neck rotation with local compression at the base of the tongue and submandibular gland can also lead to the occlusion of Wharton's duct, leading to sialoadenitis. Intraoperative neuromonitoring with cranial nerve stimulation was used. The intraoperative muscle contractions may have caused a displacement of the tube or oral packing. The surprising gradual enlargement of the submandibular gland postoperatively can be explained by reperfusion. 

The management of sialoadenitis focuses on preventing the need for invasive airway interventions. Corticosteroids are often used, although their beneficial effects have not been conclusively proven. Other conservative measures, such as sialogogues (foods that stimulate salivary flow), massage, analgesics, and intravenous hydration, are considered more beneficial [11].

## Conclusions

This case highlights a rare occurrence of macroglossia and sialoadenitis following cerebellopontine angle surgery due to patient positioning and pressure from the endotracheal tube. It emphasizes the significance of precise patient positioning, tube placement and oral packing during posterior fossa surgeries to prevent these complications.

Prolonged duration, neck (latero) flexion and rotation and local compression greatly contribute to this uncommon complication. It is mandatory to check the venous drainage of a patient in a rotated and flexed position of the neck. Especially when using armored endotracheal tubes, oral packing or intra-operative neuromonitoring, local pressure in the oral cavity on the base of the tongue but also on the surrounding mucosa should be checked regularly during surgery. Extra attention should be given to manipulation of the tube after initial placement. The tube should preferably be fixated on the ipsilateral side of the craniotomy, thereby diminishing the risk of increased local compression on the opposite side. Sialoadenitis, leading to gland enlargement, can be managed conservatively with sialogogues, massage, analgesics, and hydration to porevent deterioration. Surgical teams should be aware of these rare yet serious complications, necessitating careful attention to prevent airway compromise. Further research is needed to understand these complications' mechanisms and establish effective preventive measures.
